# High Thermal Stability Apatite Phosphors Ca_2_La_8_(SiO_4_)_6_O_2_:Dy^3+^/Sm^3+^ for White Light Emission: Synthesis, Structure, Luminescence Properties and Energy Transfer

**DOI:** 10.1038/s41598-019-51915-1

**Published:** 2019-10-29

**Authors:** Ning Liu, Lefu Mei, Libing Liao, Jie Fu, Dan Yang

**Affiliations:** Beijing Key Laboratory of Materials Utilization of Nonmetallic Minerals and Solid Wastes, National Laboratory of Mineral Materials, School of Materials Science and Technology, China University of Geosciences Beijing, Beijing, 100083 China

**Keywords:** Lasers, LEDs and light sources, Optical physics

## Abstract

What ideal w-LED phosphors always aim to do is to achieve a single phase near-sunlight emission phosphor simultaneously with both high luminescence efficiency and high thermal stability at operation temperature. And It is well known that apatite compound phosphors are one of the most promising optical materials to realize those above because of their unique structure enhanced luminescence properties and thermal stability. Here, we synthesized a co-doped single phase apatite phosphors Ca_2_La_8_(SiO_4_)_6_O_2_:Dy^3+^/Sm^3+^ (CLSO:Dy^3+^/Sm^3+^) for white light emission, which was provided with excellent thermal stability and of which luminescence intensity at 150 °C still was 92 percentage of that at room temperature. Moreover, X-ray diffraction technique, Fourier transform infrared spectroscopy, scanning electron microscope were employed to characterization of phase structure and morphology, and consequently pure apatite structure and gravel-like morphology of phosphors were proved. Analysis of photoluminescence spectra indicated that concentration quenching effect exist in single-doped CLSO:Dy^3+^ phosphors owing to dipole-dipole interaction between Dy^3+^ ions. It is revealed that maybe exist Dy^3+^ ↔ Sm^3+^ bilateral non-radiative energy transfer processes in Dy^3+^/Sm^3+^ co-doped CLSO system by PL spectra and decay curves. And variation of Sm^3+^ ion concentration can control color emission, namely CIE chromaticity coordinates and correlated color temperature, finally to achieve white light emission (0.309,0.309) with CCT 6848 K, able to be a potential candidate for commercial lighting applications.

## Introduction

At present, LEDs which can convert power into light via the electrons and holes recombination radiating visible light have been integrated into every aspect of our lives and works. For instance, colorful LEDs are applied to backlight panel for displays and projectors, or as a kind of interior decorative material; white emission LEDs (w-LEDs) are widely used in indoor and outdoor illumination for replacing traditional incandescent due to their excellent characteristics: high luminescent efficiency, brightness, lower power consumption, long operation time, lower manufacture costs, high chemical stability and eco-friendly features etc^1–3^. So far, there have been two kinds of method to get white light emission. One is that combination GaN/InGaN blue ships with YAG:Ce^3+^ yellow emission phosphors, in which white light is composed of blue light and yellow light with cold color temperature owing to lack of red emission^4,5^. The other one is based on UV/n-UV chips triple color (red, green and blue) compounds white emission, of which color rendering index is better than the former, but trade-off luminescent efficiency, different decay situations, complex assembly processes and costs expensive are primary reasons for restricting its development^6,7^. Therefore, the development of a single phase phosphors with white light emission turns into a solution.

Apatite, chemical formula is M_10_[TO_4_]_6_Z_2_, where M stands cation site with +1, +2 or +3 charges (can be occupied by K^+^, Na^+^, Ca^2+^, Sr^2+^, Ba^2+^, Pb^2+^, Mn^2+^, La^3+^, Y^3+^, Ce^3+^ etc.), and [TO_4_] represents anion group (can be replaced by [SiO_4_], [PO_4_], [GeO_4_], [MnO_4_], [VO_4_], [AsO_4_], [SO_4_] etc.), and Z is anion with −1 or −2 charges generally being O^2−^, OH^−^ and halogen ions^8,9^. Apatite phase possesses complicated structure, in which exists two kinds of independent M cation sites where [MO_9_] nine-fold coordinated polyhedron with C_3_ point symmetry and [MO_6_Z] seven-fold coordinated polyhedron with C_S_ point asymmetry, contributing to achieve different kinds emission of the same rare earth ion^10,11^. Until now, a several of apatite type phosphors with white light emission were reported, such as Ca_9_La(PO_4_)_5_(SiO_4_)F_2_:Dy^3+^ ^12^, Ba_10_(PO_4_)_6_O:Eu^2+^,Tb^3+^/Li^+^ ^13^, Mg_2_Y_8_(SiO_4_)_6_O_2_:Ce^3+^/Mn^2+^/Tb^3+^ ^14^, Ca_5_(PO_4_)_3_Cl:Dy^3+^,Li^+^/Eu^3+^ ^15^ etc. Recently, rare earth element dysprosium has been given intensively attentions and investigated in terms of their unique photoluminescence properties in luminescent materials. Dy^3+^ ion single-doped phosphors can emit near white light, and need to combinate red emission to get better white light emission, such as Sm^3+^, Eu^3+^, Mn^2+^ etc^16–18^.

Here, we synthesized Ca_2_La_8_(SiO_4_)_6_O_2_:Dy^3+^/Sm^3+^ phosphors with pure apatite structure for the first time. Traditional solidstate method was employed there. And crystallographic structure and morphology were characterized by using XRD, SEM, FT-IR, while photoluminescence spectra, quenching effect, energy transfer effect and fluorescence lifetimes were measured and analyzed. In addition, temperature dependent spectra also were measured, which indicated excellent thermal stability of 8% emission intensity decrease at 150 °C. The optimal white light emission at (0.309,0.309) with CCT 6848 K, belonging to cold white light, could be suitable applied in commercial w-LEDs application.

## Experimental

### Materials and synthesis

The solid state method was employed in synthesis of a series of Ca_2_La_8-x-y_(SiO_4_)_6_O_2_:xDy^3+^/ySm^3+^ phosphors, and the chemicals CaCO_3_, La_2_O_3_, SiO_2_, Dy_2_O_3_, Sm_2_O_3_ all are analytic grade purity and purchased by Aladdin Industrial Corporation. Typically, as synthesis of Ca_2_La_7.6_(SiO_4_)_6_O_2_:0.20Dy^3+^/0.20Sm^3+^ phosphors, marked as CLSO:0.20Dy^3+^/0.20Sm^3+^, firstly weighing and mixing raw materials with stoichiometric ratio and then grinding for nearly 10 min at agate mortar. Next the mixture was placed into an alumina crucible and pre-sintered at 1000 °C for 1 h and annealed at 1500 °C for 4 h. Finally, as-synthesized samples naturally cooled to room temperature and ground into powder for measurement.

### Measurement and characterization

The XRD patterns of all as-synthesized samples were measured by X-ray powder diffractometer (D8 Advance, Bruker Corporation, Germany) with Cu-Kα radiation λ = 0.15406 nm under the condition of 40 KV and 30 mA, and the range from 10° to 80°. The SEM images were identified by high resolution field emission scanning electron microscope (JSM-7001F). The FT-IR patterns were identified on Fourier transform infrared spectrometer (Spectrum 100, Perkinelmer). The PL and PLE spectra at room temperature were recorded by fluorescence spectrometer (Hitachi F-4600) with a excitation resource xenon lamp (400 V, 150 W), and a 400 nm cut-off filter was used. The decay curves were measured by a spectro-fluorometer (Horiba, Jobin-Yvon TBXPS). Above of measurements are under room temperature. The temperature-dependence spectra were recorded on a spectro-fluorometer (Horiba, Jobin-Yvon Fluorolog-3 FL3-21), combined with a self-made heating attachment and a computer-controlled electric furnace (Tianjin Orient KOJI Co. Ltd, TAP-02).

## Results and Discussion

### Structure and morphology

**Figure 1:**
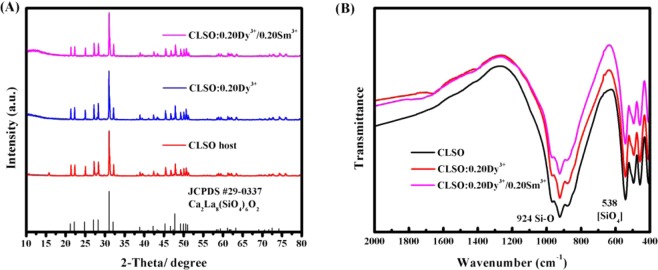
(**A**) The XRD patterns of CLSO host and CLSO:0.20Dy^3+^, CLSO:0.20Dy^3+^/0.20Sm^3+^ samples and the standard card JCPDS #29-0337 Ca_2_La_8_(SiO_4_)_6_O_2_ shown as a reference. (**B**) The FT-IR spectroscopy of as-synthesized CLSO host, CLSO:0.20Dy^3+^, CLSO:0.20Dy^3+^/0.20Sm^3+^ samples.

Figure 1(A) shows the XRD patterns of CLSO host and CLSO:0.20Dy^3+^, CLSO:0.20Dy^3+^/0.20Sm^3+^ phosphors. As observed, there are similar XRD patterns for the CLSO host and doped samples. And no impurity phase peaks appear, which indicates that rare earth ions substitute the host lattice causing little changes in crystal structure, due to ion radii of Dy^3+^ (r = 0.97 Å for CN = 7 and r = 1.08 Å for CN = 9)and Sm^3+^ (r = 1.02 Å for CN = 7 and r = 1.13 Å for CN = 9)are close to that of La^3+^ (r = 1.10 Å for CN = 7 and r = 1.22 Å for CN = 9)of the CLSO host^12,19,20^. JADE6.5 software was utilized to analyze these XRD patterns, based on Scherrer’s equation and lattice strain theory, and analysis results, shown on Table 1, demonstrate all samples belonging to hexagonal apatite structure and space group P6_3_/m. The calculated values of CLSO host are little different from that JCPDS No.29-0337 given, and as RE^3+^ ions doped there is an irregular decrease in cell parameter and unit cell volume (more cell parameters see Supplementary Tables S1–S3), owing to in theoretical prediction impurity Dy^3+^ or Sm^3+^ ions should only occupy La^3+^ ions lattice resulting in a regular decrease as impurity RE^3+^ ions concentration increases in CLSO host crystal structure, but the actual situation exists some difference with theories. During the synthesis process, thermal diffusion being a stochastic and uncontrollable process, though ion radius of Dy^3+^ or Sm^3+^ is close to that of La^3+^ and more suitable to substitute La^3+^, there still a few part of Ca^2+^ are replaced by impurity ions and will generate a Ca^2+^ vacancy when an impurity RE^3+^ ion with +3 charges occupies a Ca^2+^ ion lattice point with +2 charges, bringing about irregular lattice distortion.

**Table 1:** Space group, cell parameters, and unit cell volume of compounds.

Compound	Space Group	a(b)/Å	c/Å	V/Å^3^
JCPDS #29-0337	P6_3_/m	9.651	7.151	576.822
CLSO	P6_3_/m	9.632	7.186	577.365
CLSO:0.20Dy^3+^	P6_3_/m	9.619	7.137	571.881
CLSO:0.20Dy^3+^/0.20Sm^3+^	P6_3_/m	9.633	7.111	571.458

**Figure 2:**
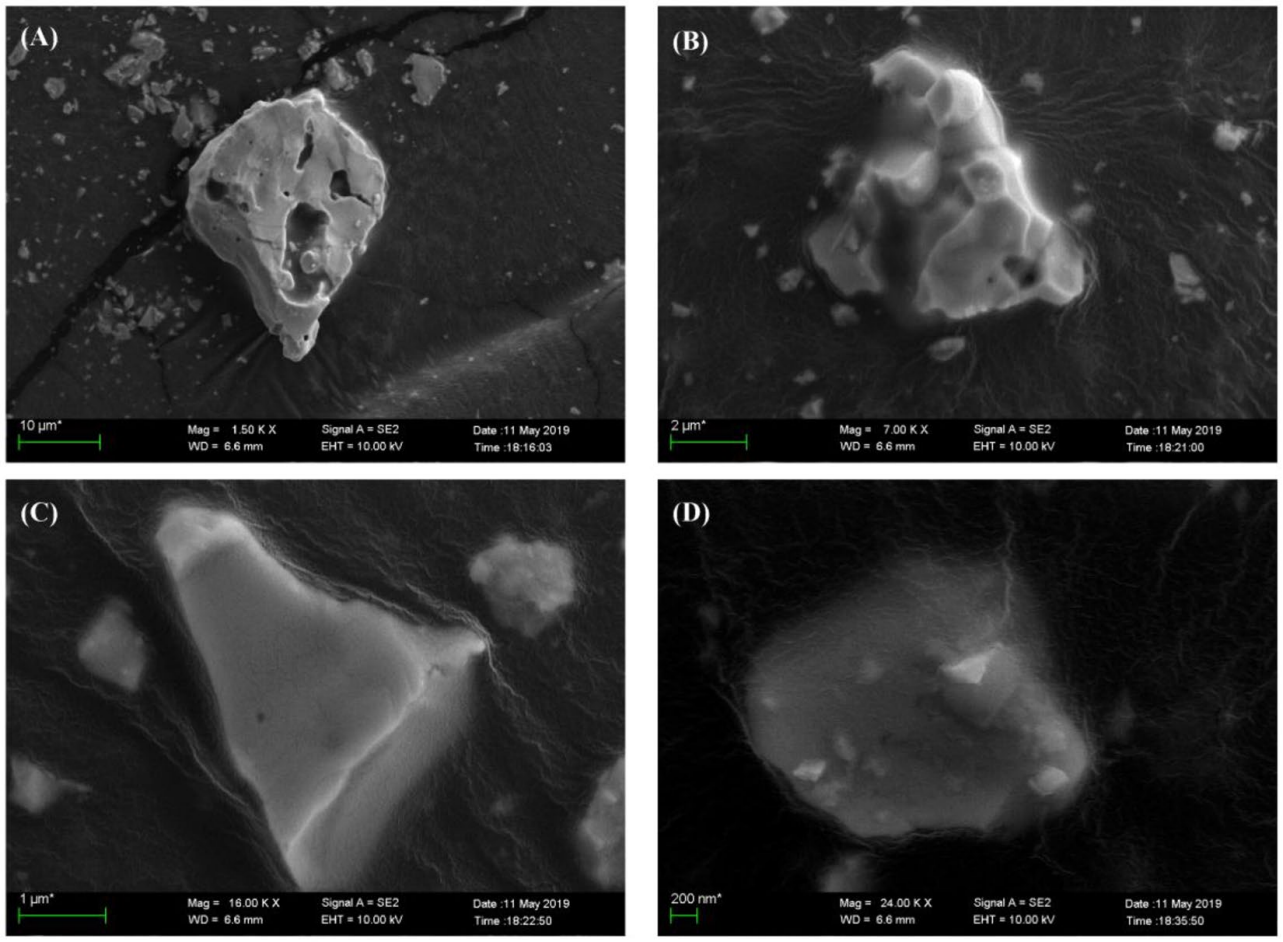
SEM images of CLSO:0.20Dy^3+^/0.20Sm^3+^ with different magnification.

FT-IR pattern of the CLSO host and Dy^3+^, Sm^3+^ doped phosphors are shown on Fig. 1(B). As shown, the doped phosphors’ curves are slightly different from the CLSO host, with same absorption peaks at 924 cm^−1^ and 600–400 cm^−1^, indicated that dopant will generate negligible influence in term of host crystal structure, completely consistent with XRD analysis results mentioned above. According to the literature^21^, [SiO_4_] tetrahedron vibration absorption peaks located on 1100–900 cm^−1^ and 600–400 cm^−1^, respectively correspond to the asymmetric Si-O bond stretching modes and the [SiO_4_] silica tetrahedron bending modes. In this host, the asymmetric stretching of Si-O bond produces an absorption peak of which wavenumber is 924 cm^−1^, and a multiple absorption peak at 538–400 cm^−1^ should be assigned to silica tetrahedron bending. Figure 2 shows the SEM images of CLSO:CLSO:0.20Dy^3+^/0.20Sm^3+^ sample, and from picture A to picture D magnification are 1.5 K X, 7.00 K X, 16.00 K X and 24.00 K X, respectively. As we observed in images, sample particles have no fixed shape, belonging to gravel-like morphology with particle size from 1 μm to 10 μm.

### Photoluminescence spectra

**Figure 3:**
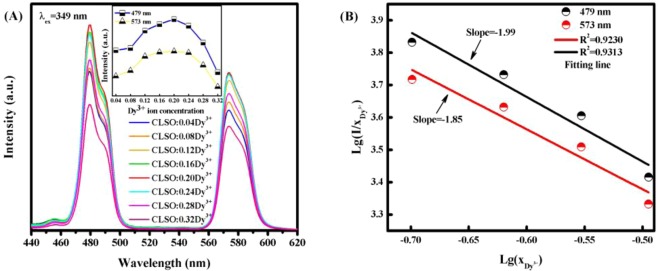
(**A**) The PL spectra of CLSO:xDy^3+^ (x = 0.04–0.32) phosphors on Dy^3+^ doping content (x), inserted graph plots intensity at 479 nm and 573 nm versus Dy^3+^ doping concentration. (**B**) The fitting line of Lg(I/x) versus Lg(x) in CLSO:xDy^3+^ phosphors.

The PL spectra of CLSO:xDy^3+^ (x = 0.04, 0.08, 0.12, 0.16, 0.20, 0.24, 0.28 and 0.32) excited at 349 nm are presented in Fig. 3(A), which demonstrate that intensity of emission at 479 nm and 573 nm depend on Dy^3+^ doping concentration. Two emission peaks shown on Fig. 3(A), of which peak center at 479 nm and 573 nm correspond to ^4^F_9/2_ − ^6^H_15/2_ and ^4^F_9/2_ − ^6^H_13/2_ transition respectively, and there is a very low intensity emission at 664 nm corresponding to ^4^F_9/2_ − ^6^H_11/2_ transition not shown^22^.It is clearly found that the emission intensity at 479 nm and 573 nm both increase first, and when 0.20 concentration of Dy^3+^ them reache a maximum, and then decrease as the concentration increasing unceasingly owing to the concentration quenching effect^23^. According to Van Uitert reported^24^, the electric multi-polar interaction type dominating energy transfer between adjacent Dy^3+^ ions of sensitizers and activators, could be estimated by using following Eq. (1):

1 $$\frac{I}{x}=k{[1+\beta {(x)}^{\theta /3}]}^{-1}$$

where x refers to the activator Dy^3+^ ion concentration, I/x represents the emission intensity per activator concentration, k and β are constants for host lattice^25^. Dipole-dipole, dipole-quadrupole, quadrupole-quadrupole interactions respectively correspond with the values of θ = 6, 8, 10. The above Eq. (1) could equivalently transform into Eq. (2), as follows:

2 $$aLg(\frac{I}{x})=-\,\frac{\theta }{3}Lg(x)+R$$

where R is a constant related to k and β. Figure 3(B) shows the fitting line of Lg(I/x) versus Lg(x) in CLSO:xDy^3+^ phosphors with different wavelength at 479 nm and 573 nm respectively beyond the quenching concentration. It is clearly found that the fitting curves of Lg(I/x) versus Lg(x) are well matched with relatively linear correlation and the slopes were confirmed to be −1.99 and −1.85, corresponding with ^4^F_9/2_ − ^6^H_15/2_ and ^4^F_9/2_ − ^6^H_13/2_ transition respectively. Therefore, θ which equals the value of slope multiplied by −3, and are 5.97 and 5.55 respectively. Both of the θ values obtained are closest to 6, meaning that dipole-dipole interaction between Dy^3+^ ions dominants in energy transfer process, consistent with Liu *et.al*.^12^ Sm^3+^ doped CLSO phosphors also have been studied (see Supplementary Figs S1 and S2), and dipole-dipole interaction is proved.

**Figure 4:**
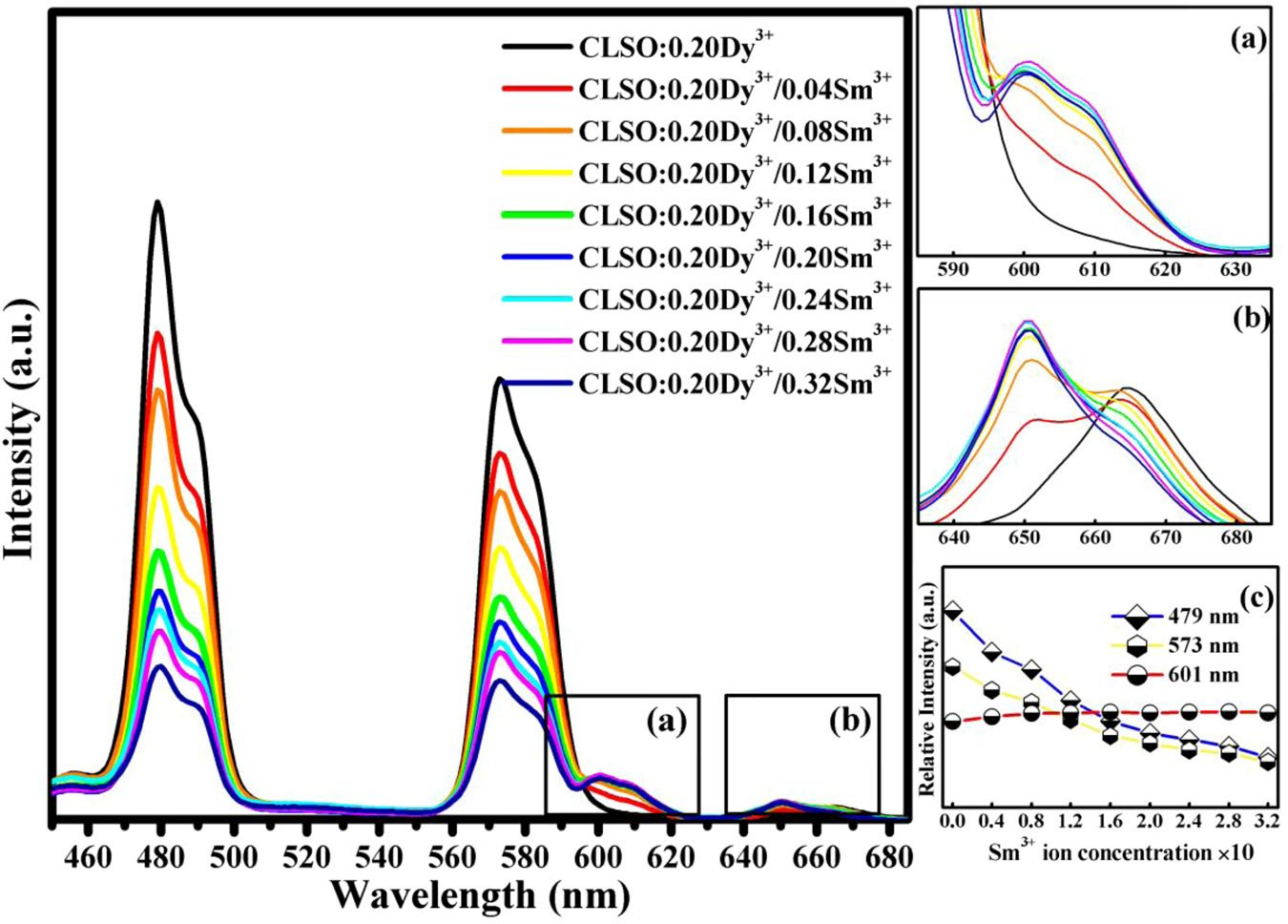
The PL spectra of CLSO:0.20Dy^3+^/ySm^3+^ (y = 0 − 0.32)phosphors, (**a**,**b**) are partial enlargement of the spectra, (**c**) depicts emission intensity variation at 479 nm, 573 nm and 601 nm in pace with Sm^3+^ ion concentration increasing.

In addition, CLSO:0.20Dy^3+^/ySm^3+^ phosphors PL spectra, y varying from 0 to 0.32, were measured, and shown on Fig. 4. It can be clearly observed that with Sm^3+^ ion doping concentration increasing, the characteristic peaks intensity of Dy^3+^ at 479 nm and 573 nm both show gradually decreasing due to existence of Dy^3+^ → Sm^3+^ energy transfer in Dy^3+^/Sm^3+^ co-doped CLSO phosphors system. However, peak at 601 nm which is the characteristic emission of Sm^3+^ ion in CLSO system, do not exhibit an obvious increase (Fig. 4a,c)with its concentration increasing that is different from Sm^3+^ single doped CLSO spectra (see Fig. S1). And there two hypotheses proposed, the first one is that absorption and emission efficiency of Dy^3+^ at 365 nm wavelength both are much higher than those of Sm^3+^ in CLSO system, the other one is that in the system maybe exist Sm^3+^ → Dy^3+^ and Sm^3+^ → Sm^3+^ non-radiative energy transfer phenomenon too.

### Energy transfer

**Figure 5:**
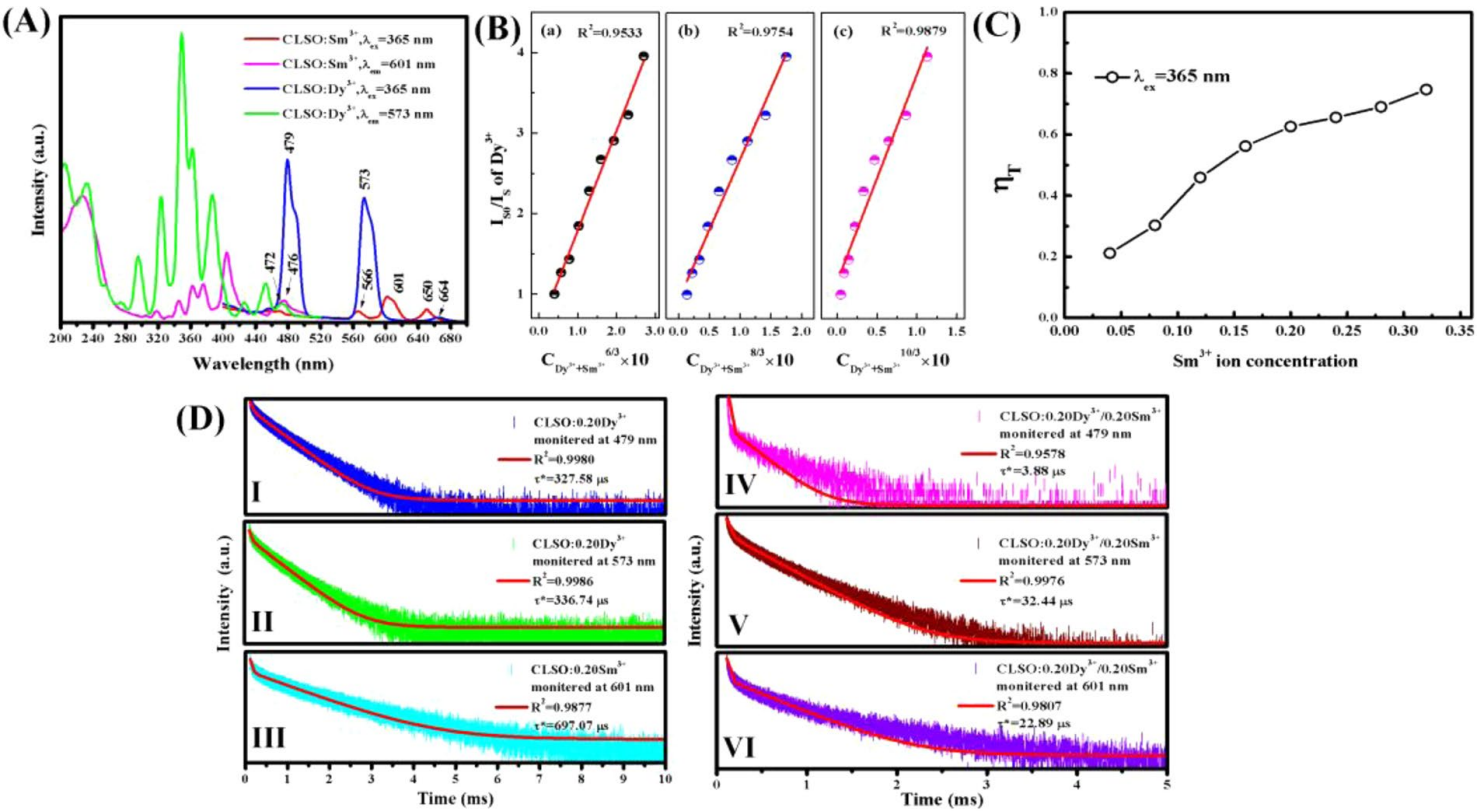
(**A**) The PL and PLE spectra of CLSO:Dy^3+^ and CLSO:Sm^3+^ phosphors. (**B**) The linear fitting of I_SO_/I_S_ of Dy^3+^ versus C^n/3^ in CLSO:0.20Dy^3+^/ySm^3+^ (y = 0–0.32) phosphors. (**C**) The plotting of η_T_ versus Sm^3+^ ion concentration. (**D**) Decay curves of Dy^3+^ in CLSO:0.20Dy^3+^ phosphor excited at 365 nm monitored at 479 nm (I) and 573 nm (II); (III) decay curve of Sm^3+^ in CLSO:0.20Sm^3+^ phosphor excited at 365 nm monitored at 601; decay curves of CLSO:0.20Dy^3+^/0.20Sm^3+^ phosphor excited at 365 nm monitored at 479 nm (IV), 573 nm (V) and 601 nm (VI).

The spectra appear variation phenomenon along with Sm^3+^ ion doping concentration increasing because energy transfer effect dominants. To further understand the energy transfer processes, the PL and PLE spectra, interaction type, critical distance and energy transfer efficiency all were investigated. Figure 5(A) shows the PL spectra of single doped CLSO:Dy^3+^ and CLSO:Sm^3+^ phosphors at 365 nm excitation, and the PLE spectra of them monitored at 573 nm and 601 nm respectively. emission peaks center of CLSO:Sm^3+^ appear at 566 nm, 601 nm and 650 nm caused by the electronic energy level transition of ^4^G_5/2_ → ^6^H_5/2_, ^4^G_5/2_ → ^6^H_7/2_ and ^4^G_5/2_ → ^6^H_9/2_, respectively^22^. From Fig. 5(A), it can be seen that the PL spectrum (blue line) at 479 nm of Dy^3+^ overlap with the PLE spectrum (purple line) at 476 nm of Sm^3+^ proving existence of Dy^3+^ → Sm^3+^ energy transfer, and also overlap with the green line PLE spectrum monitored at 579 nm of Dy^3+^ revealing Dy^3+^ → Dy^3+^ energy transfer. Emission peak at 566 nm and emission peak at 579 nm overlapped is the cause of characteristic peak at 566 nm not shown on PL spectra of Fig. 4.

According to the Dexter’s multipolar interaction energy transfer formula^26^, as follows:

3 $$\frac{{\eta }_{S0}}{{\eta }_{S}}\propto {C}^{n/3}$$

where η_S0_, η_S_ are the luminescence quantum efficiencies of Dy^3+^ in the absence and presence of Sm^3+^ respectively, and C stands the total concentration of doping ions, which equals 0.20(Dy^3+^) + y(Sm^3+^). The values of n = 6, 8, 10 correspond with dipole-dipole, dipole-quadrupole, quadrupole-quadrupole interactions, respectively. Owing to the values of η_S0_ and η_S_ are hard to measure and η_S0_/η_S_ approximately equals I_S0_/I_S_, which stands emission intensity of Dy^3+^ with/without Sm^3+^ ion existence, formula (3) can be convert into following formula:

4 $$\frac{{I}_{S0}}{{I}_{S}}\propto {C}^{n/3}$$

The relationship between I_S0_/I_S_ and C^n/3^ based on formula (4) are plotted in Fig. 5(B). It can be found that when n value takes 10, the value of linear fitting coefficient R^2^ is biggest and linear behavior is best, therefore it reveals Dy^3+^ → Sm^3+^ energy transfer via quadrupole-quadrupole interaction mechanism, which is consistent with others’ previous investigation^27^.

With the increase of Sm^3+^ dopant content, the emission spectra intensity of Sm^3+^ activators were observed to increase slightly whereas PL spectra intensity of Dy^3+^ sensitizers simultaneously occur significantly decreasing. The energy transfer efficiency η_T_ from Dy^3+^ to Sm^3+^ can be calculated by following equation^28^:

5 $${\eta }_{T}=1-\frac{{I}_{S}}{{I}_{S0}}$$

Figure 5(C) shows the plotting of η_T_ versus Sm^3+^ ion concentration. As it shown, the energy transfer efficiency increases in pace with Sm^3+^ ion concentration increasing, and When Sm^3+^ ion concentration is 0.32, the efficiency can reach to 74.72%. and it can be estimated that the critical concentration when energy transfer efficiency is 50%, approximately equals 0.336.

According to the Dexter-Schulman theory the critical concentration will be higher if the energy transfer probability is lower. And the energy transfer probability depends on the distance between sensitizers and activators ions. Therefore, the Dy^3+^ → Sm^3+^ energy transfer probability depending on the distance between Dy^3+^ and Sm^3+^, on the basis of Blasse’s expression^29^, the critical distance (R_C_) of energy transfer can be calculated by Eq. (6), as follows:

6 $${R}_{{\rm{c}}}=2{(\frac{3V}{4\pi {x}_{{\rm{c}}}{\rm{N}}})}^{1/3}$$

where V stands the volume of the crystallographic unit cell, and x_c_ the critical concentration, and N the number of lattice sites which can be occupied by dopant ions in the unit cell and N = 1. Substituting V, x_c_ and N into Eq. (6), it is found that the critical distance R_C_ equals 14.809 Å in CLSO:0.20Dy^3+^/0.20Sm^3+^ phosphors. And similar values are found in other systems^27,30^. Therefore, when distance between rare earth ions R < 6 Å, there exchange interaction dominants in energy transfer processes^31^ while quadrupole-quadrupole interaction contributes to those processes when 6 Å < R < 14.809 Å in CLSO:0.20Dy^3+^/0.20Sm^3+^ phosphor.

### Decay curves

To further understand the de-excitation and Dy^3+^ → Sm^3+^ energy transform processes, decay curves of CLSO:Dy^3+^, CLSO:Sm^3+^ and co-doped CLSO:Dy^3+^/Sm^3+^ phosphors are measured for ^4^F_9/2_ level of Dy^3+^ ions and ^4^G_5/2_ level of Sm^3+^ ions, and shown on Fig. 5(D). Red lines all are fitting lines and fitting index R^2^ value also are shown on Fig. 5(D). All samples are excited at 365 nm wavelength with different monitored wavelength, and it can be found that all of decay curves can be successfully fitted with a typical second order exponential decay equation^32^ as follows:

7 $$I({\rm{t}})={I}_{0}+{A}_{1}\exp (\,-\,{\rm{t}}/{\tau }_{1})+{A}_{2}\exp (\,-\,{\rm{t}}/{\tau }_{2})$$

where I(t) represents at time t the luminescence intensity and I_0_ is the initial luminescence intensity, A_1_ and A_2_ are decay constants, τ_1_ and τ_2_ respectively stand slow and rapid lifetimes for exponential components. Besides, the effective time (τ^*^) can be calculated as following equation:

8 $${\tau }^{\ast }=({A}_{1}{\tau }_{1}^{2}+{A}_{2}{\tau }_{2}^{2})/({A}_{1}{\tau }_{1}+{A}_{2}{\tau }_{2})$$

Hereafter, the effective lifetimes of ^4^F_9/2_ level of Dy^3+^ in CLSO:0.20Dy^3+^ phosphor are calculated to be 327.58 μs (479 nm) and 336.74 μs (573 nm), and those in CLSO:0.20Dy^3+^/0.20Sm^3+^ phosphor are calculated to be 3.88 μs (479 nm) and 32.44 μs (573 nm); and the effective lifetime of ^4^G_5/2_ level of Sm^3+^ in CLSO:0.20Sm^3+^ phosphor at 601 nm is found to be 697.07 μs and that in CLSO:0.20Dy^3+^/0.20Sm^3+^ phosphor is 22.89 μs. It can be obviously observed that the lifetimes of Dy^3+^ and Sm^3+^ have a sharp decrease, considering that the distance between Dy^3+^ and Dy^3+^, Dy^3+^ and Sm^3+^, Sm^3+^ and Sm^3+^ decreases with dopant concentration increasing, causing the probability of energy transfer to luminescent killer sites rising. The lifetime decrease of Dy^3+^ in co-doped phosphor owing to existing Dy^3+^ → Sm^3+^ non-radiative energy transfer because of the overlap between peak at 479 nm of emission spectra of Dy^3+^ and peak at 476 nm of excitation spectra of Sm^3+^, interestingly, the lifetime of Sm^3+^ also appear a sharp decline for the reason that there may exist Dy^3+^ ↔ Sm^3+^ bilateral non-radiative energy transfer, consistent with the second hypothesis mentioned above in spectra section.

### Temperature dependent PL spectra

**Figure 6:**
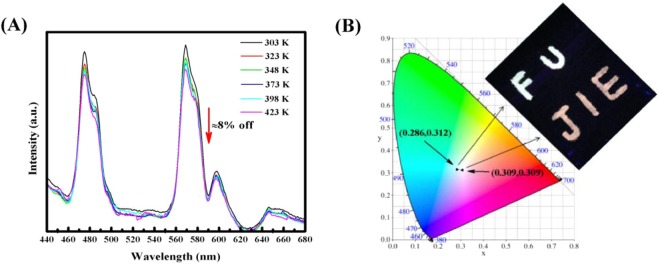
(**A**) Temperature dependent PL spectra of CLSO:0.20Dy^3+^/0.20Sm^3+^ phosphor in the range of 303–423 K. (**B**) The chromaticity diagram of CLSO:0.20Dy^3+^ (0.286,0.312) and CLSO:0.20Dy^3+^/0.20Sm^3+^ (0.309,0.309) phosphors, and digital photograph shows that “FU” and “JIE” are spelled using CLSO:0.20Dy^3+^ and CLSO:0.20Dy^3+^/0.20Sm^3+^ phosphors at 365 nm excitation respectively.

To further investigate the possible practical application under high power condition, the temperature dependent photoluminescence spectra of CLSO:0.20Dy^3+^/0.20Sm^3+^ phosphor ranging from 303 K to 418 K have been measured at excitation wavelength of 365 nm shown on Fig. 6(A). With temperature increasing from room temperature 303 K to 423 K, it depicts that there is no change occurring in terms of the position and shape of the emission spectra while just the intensity of the emission spectrum decreases. When temperature turns up to 423 K the emission intensity decreases about 8%, compared with that at room temperature 303 K,, which indicates that the CLSO:0.20Dy^3+^/0.20Sm^3+^ phosphor exhibits excellent thermal stability for potential w-LED application.

### CIE color coordinate and correlated color temperature

The the Commission Internationale de L’Eclairage (CIE) color coordinate and correlated color temperature (CCT) are two important indicators directly inspecting accurate color emission, color purity and its temperature of phosphors. These CIE chromaticity coordinates were calculated based on their PL spectrum, and the results are shown on Table 2 with CCT. The correlated color temperature can be estimated by following McCamy empirical formula^27,33^:

9 $${\rm{CCT}}=-\,{\rm{449}}{n}^{3}+3525{n}^{2}-6823n+5520.33$$

where n equals (x − x_e_)/(y − y_e_), and the chromaticity epicenter (x_e_, y_e_) is (0.3320,0.1858). All of CCT of CLSO apatite phosphors are over 5000 K, which is boundary temperature, CCT of white light emission less than 5000 K named warn white light used for household lighting or appliances and conversely clod white light more suitable for commercial application for lighting purposes. From Table 2, it can be observed that CLSO:0.20Dy^3+^/0.20Sm^3+^ apatite phosphor exhibits better chromaticity coordinate than others at 365 nm excitation, being closet to the ideal white light emission point (0.333,0.333). Figure 6(B) shows the chromaticity diagram of CLSO:0.20Dy^3+^ and CLSO:0.20Dy^3+^/0.20Sm^3+^ phosphors excited at 365 nm, which are consistent with display light emission in the insert digital photograph, of which “FU” spelled by CLSO:0.20Dy^3+^ phosphor and “JIE” spelled by CLSO:0.20Dy^3+^/0.20Sm^3+^ phosphors at 365 nm excitation as well. Those emission characteristics our CLSO:Dy^3+^/Sm^3+^ phosphors demonstrated indicate that they can use for commercial w-LEDs application.

**Table 2:** Table of the comparison of the CIE color coordinates (x,y) and CCT (K) of CLSO:0.20Dy^3+^/ySm^3+^ (y = 0–0.32) phosphors excited at 365 nm.

Samples	CIE coordinates (x,y)	CCT (K)
CLSO:0.20Dy^3+^	(0.286, 0.312)	8419
CLSO:0.20Dy^3+^/0.04Sm^3+^	(0.299, 0.320)	7419
CLSO:0.20Dy^3+^/0.08Sm^3+^	(0.295, 0.308)	7916
CLSO:0.20Dy^3+^/0.12Sm^3+^	(0.304, 0.311)	7227
CLSO:0.20Dy^3+^/0.16Sm^3+^	(0.304, 0.305)	7266
CLSO:0.20Dy^3+^/0.20Sm^3+^	(0.309, 0.309)	6848
CLSO:0.20Dy^3+^/0.24Sm^3+^	(0.294, 0.290)	8441
CLSO:0.20Dy^3+^/0.28Sm^3+^	(0.308, 0.302)	7064
CLSO:0.20Dy^3+^/0.32Sm^3+^	(0.301, 0.291)	7830

## Conclusion

In this article, a series of CLSO:Dy^3+^/Sm^3+^ were synthesized by high temperature solid state method, and all as-synthesized phosphors are pure apatite structure and gravel-like morphology with particle size ranging 1–10 μm. In single doped CLSO:Dy^3+^ phosphors, the characteristic peaks of Dy^3+^ occur at 479 nm, 573 nm and 664 nm due to ^4^F_9/2_ − ^6^H_15/2_, ^4^F_9/2_ − ^6^H_13/2_ and ^4^F_9/2_ − ^6^H_11/2_ transition, respectively. Moreover, Dy^3+^ − Dy^3+^ dipole-dipole interaction primarily contributes to emission quenching effect and quenching concentration at 0.20 Dy^3+^ ion concentration. In co-doped CLSO:Dy^3+^/Sm^3+^ phosphors the luminescence spectra confirmed that existence of Dy^3+^ → Sm^3+^ energy transfer phenomenon via quadrupole-quadrupole interaction with the critical distance 14.809 Å. Energy transfer efficiency can up to 74.72% with Sm^3+^ ion doping concentration increasing. Decay curves reveal that Dy^3+^ → Sm^3+^ energy transfer result in lifetimes of ^4^F_9/2_ level of Dy^3+^ having a sharp decrease, and it also occur a sharp lifetime decay of ^4^G_5/2_ level of Sm^3+^ due to there may exists Dy^3+^ ↔ Sm^3+^ bilateral non-radiative energy transfer. Our CLSO:0.20Dy^3+^/0.20Sm^3+^ apatite phosphor exhibits cold white light emission with CIE chromaticity coordinate (0.309,0.309) and CCT 6848 K. In addition, CLSO:0.20Dy^3+^/0.20Sm^3+^ phosphor demonstrates excellent thermal stability and at 423 K emission intensity still is 92% of that at room temperature. These characteristics reveal CLSO:Dy^3+^/Sm^3+^ can be a potential candidate for commercial w-LEDs devices.

## Supplementary information

Supplementary Information
