# Supplementary material for: High Thermal Stability Apatite Phosphors Ca2La8(SiO4)6O2:Dy3+/Sm3+ for White Light Emission: Synthesis, Structure, Luminescence Properties and Energy Transfer
Source: Sci Rep. 2019 Oct 29;9:15509. doi: 10.1038/s41598-019-51915-1 (PMC6820759; doi:10.1038/s41598-019-51915-1)
Supplement: Supplementary file 1 — Supplementary Information [file 41598_2019_51915_MOESM1_ESM.docx]

**Supplementary Information**

**High Thermal Stability Ca_2_La_8_(SiO_4_)_6_O_2_:Dy^3+^/Sm^3+^ Apatite Phosphors for White Light Emission: Synthesis, Structure, Luminescence Properties and Energy Transfer**

Ning Liu, Lefu Mei*, Libing Liao*, Jie Fu, Dan Yang

*Beijing Key Laboratory of Materials Utilization of Nonmetallic Minerals and Solid Wastes, National Laboratory of Mineral Materials, School of Materials Science and Technology, China University of Geosciences Beijing, Beijing 100083, China*

*Corresponding authors for E-mail addresses: [mlf@cugb.edu.cn](mailto:mlf@cugb.edu.cn)(L. Mei), [lbliao@cugb.edu.cn](mailto:clayl@cugb.edu.cn)(L. Liao)

Table S1 space group, cell parameters, and unit cell volume of CLSO:Dy compounds.

| Sample | Space Group | a(b)/ Å | c / Å | V / Å^3^ |
| --- | --- | --- | --- | --- |
| JCPDS #29-0337 | P6_3_/m | 9.651 | 7.151 | 576.822 |
| CLSO:0.04Dy^3+^ | P6_3_/m | 9.634 | 7.054 | 566.995 |
| CLSO:0.08Dy^3+^ | P6_3_/m | 9.628 | 7.114 | 571.105 |
| CLSO:0.12Dy^3+^ | P6_3_/m | 9.648 | 7.090 | 571.546 |
| CLSO:0.16Dy^3+^ | P6_3_/m | 9.646 | 7.113 | 573.162 |
| CLSO:0.20Dy^3+^ | P6_3_/m | 9.619 | 7.137 | 571.881 |
| CLSO:0.24Dy^3+^ | P6_3_/m | 9.619 | 7.101 | 568.997 |
| CLSO:0.28Dy^3+^ | P6_3_/m | 9.647 | 7.124 | 574.168 |
| CLSO:0.32Dy^3+^ | P6_3_/m | 9.597 | 7.100 | 566.317 |

Table S2 space group, cell parameters, and unit cell volume of CLSO:Sm compounds.

| Sample | Space Group | a(b)/ Å | c / Å | V / Å^3^ |
| --- | --- | --- | --- | --- |
| JCPDS #29-0337 | P6_3_/m | 9.651 | 7.151 | 576.822 |
| CLSO:0.04Sm^3+^ | P6_3_/m | 9.636 | 7.114 | 572.055 |
| CLSO:0.08Sm^3+^ | P6_3_/m | 9.629 | 7.115 | 571.304 |
| CLSO:0.12Sm^3+^ | P6_3_/m | 9.627 | 7.097 | 569.622 |
| CLSO:0.16Sm^3+^ | P6_3_/m | 9.609 | 7.149 | 571.652 |
| CLSO:0.20Sm^3+^ | P6_3_/m | 9.615 | 7.164 | 573.567 |
| CLSO:0.24Sm^3+^ | P6_3_/m | 9.643 | 7.124 | 573.692 |
| CLSO:0.28Sm^3+^ | P6_3_/m | 9.649 | 7.205 | 580.937 |
| CLSO:0.32Sm^3+^ | P6_3_/m | 9.652 | 7.157 | 577.425 |

Table S3 space group, cell parameters, and unit cell volume of CLSO:Dy/Sm compounds.

| Sample | Space Group | a(b)/ Å | c / Å | V / Å^3^ |
| --- | --- | --- | --- | --- |
| JCPDS #29-0337 | P6_3_/m | 9.651 | 7.151 | 576.822 |
| CLSO:0.20Dy/0.04Sm^3+^ | P6_3_/m | 9.638 | 7.073 | 568.995 |
| CLSO:0.20Dy/0.08Sm^3+^ | P6_3_/m | 9.575 | 7.043 | 559.198 |
| CLSO:0.20Dy/0.12Sm^3+^ | P6_3_/m | 9.642 | 7.115 | 572.849 |
| CLSO:0.20Dy/0.16Sm^3+^ | P6_3_/m | 9.617 | 7.155 | 573.086 |
| CLSO:0.20Dy/0.20Sm^3+^ | P6_3_/m | 9.633 | 7.111 | 571.458 |
| CLSO:0.20Dy/0.24Sm^3+^ | P6_3_/m | 9.645 | 7.119 | 573.527 |
| CLSO:0.20Dy/0.28Sm^3+^ | P6_3_/m | 9.636 | 7.114 | 572.055 |
| CLSO:0.20Dy/0.32Sm^3+^ | P6_3_/m | 9.637 | 7.118 | 572.496 |


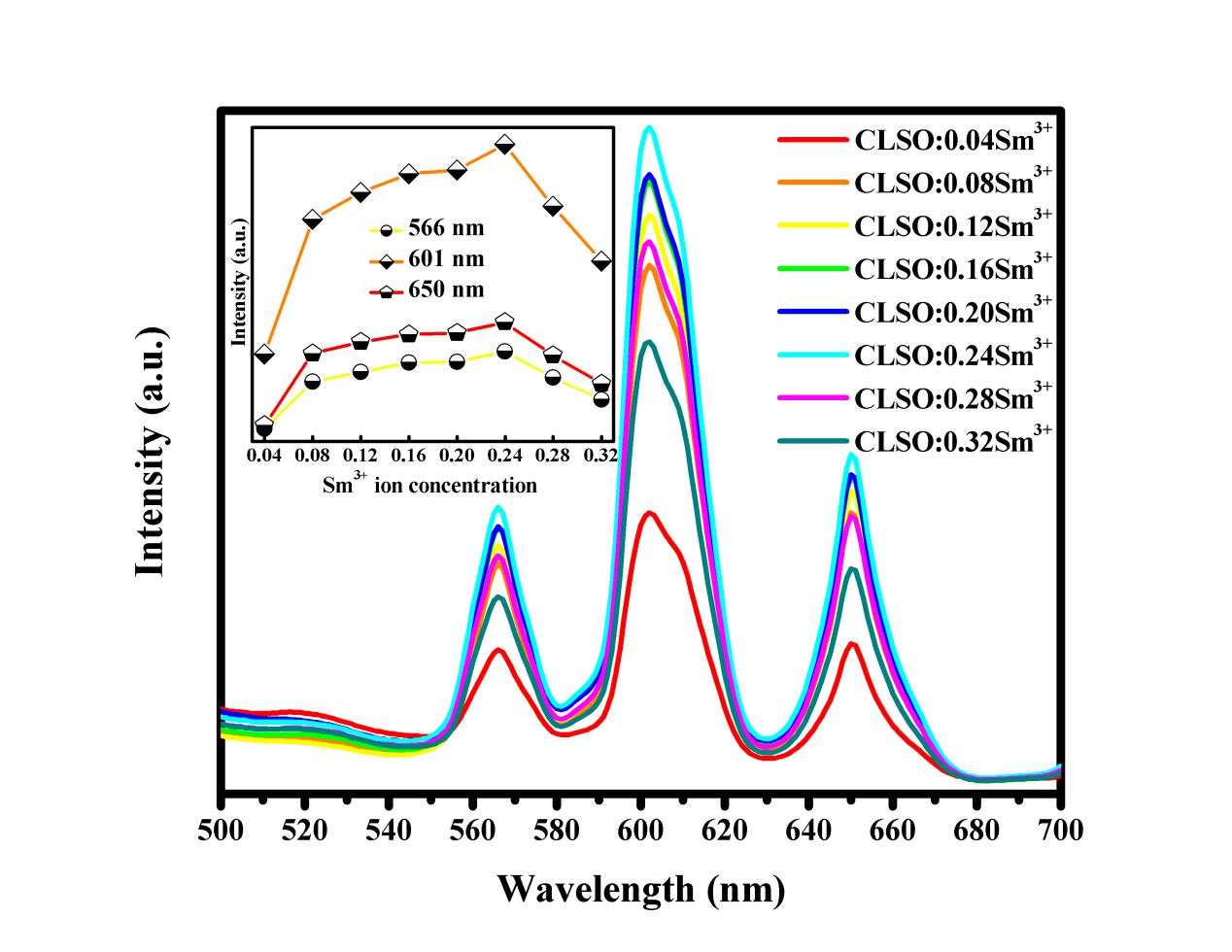


Figure S1 The PL spectra of CLSO:ySm^3+^ (y=0.04-0.32) phosphors on Sm^3+^ doping content (y), inserted graph plots intensity at 566 nm, 601 nm and 650 nm versus Sm^3+^ doping concentration.


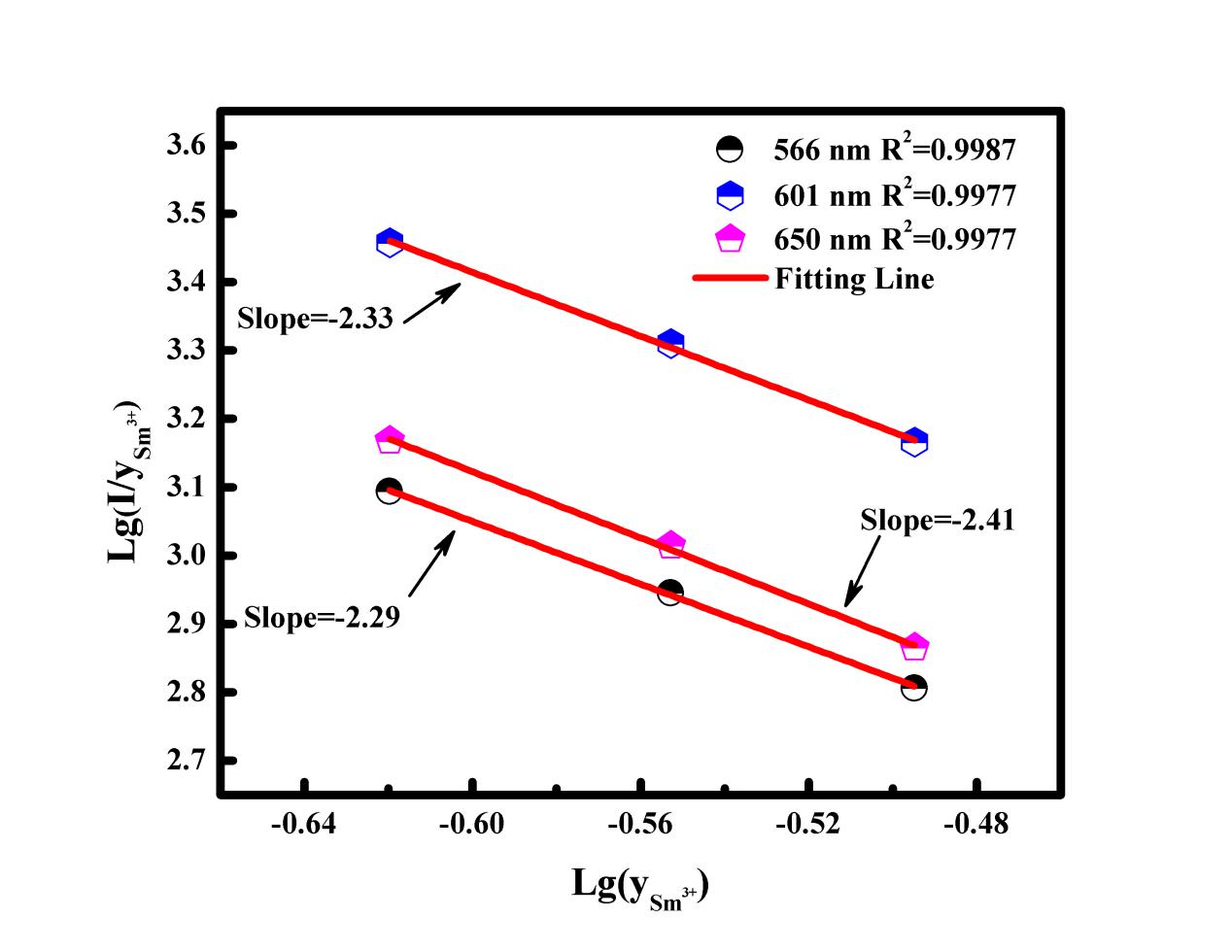


Figure S2 The fitting line of Lg(I/y) versus Lg(y) in CLSO:yDy^3+^ phosphors.
